# Asymmetrical transfer effects of cognitive bias modification: Modifying attention to threat influences interpretation of emotional ambiguity, but not vice versa

**DOI:** 10.1016/j.jbtep.2016.08.011

**Published:** 2017-03

**Authors:** J.O. Bowler, L. Hoppitt, J. Illingworth, T. Dalgleish, M. Ononaiye, G. Perez-Olivas, B. Mackintosh

**Affiliations:** a School of Psychology, University of East Anglia, UK; b Medical Research Council Cognition and Brain Sciences Unit, Cambridge, UK; c Norwich Medical School, University of East Anglia, UK

**Keywords:** Cognitive bias modification, Attention, Interpretation, Transfer effects, Anxiety

## Abstract

**Background and objectives:**

It is well established that attention bias and interpretation bias each have a key role in the development and continuation of anxiety. How the biases may interact with one another in anxiety is, however, poorly understood. Using cognitive bias modification techniques, the present study examined whether training a more positive interpretation bias or attention bias resulted in transfer of effects to the untrained cognitive domain. Differences in anxiety reactivity to a real-world stressor were also assessed.

**Methods:**

Ninety-seven first year undergraduates who had self-reported anxiety were allocated to one of four groups: attention bias training (*n* = 24), interpretation bias training (*n* = 26), control task training (*n* = 25) and no training (*n* = 22). Training was computer-based and comprised eight sessions over four weeks. Baseline and follow-up measures of attention and interpretation bias, anxiety and depression were taken.

**Results:**

A significant reduction in threat-related attention bias and an increase in positive interpretation bias occurred in the attention bias training group. The interpretation bias training group did not exhibit a significant change in attention bias, only interpretation bias. The effect of attention bias training on interpretation bias was significant as compared with the two control groups. There were no effects on self-report measures.

**Limitations:**

The extent to which interpretive training can modify attentional processing remains unclear.

**Conclusions:**

Findings support the idea that attentional training might have broad cognitive consequences, impacting downstream on interpretive bias. However, they do not fully support a common mechanism hypothesis, as interpretive training did not impact on attentional bias.

## Highlights

• Threat-related attentional and interpretive bias are known to be causal in anxiety.
• How these bias interact with each other is less well known.
• We modified both attentional and interpretive bias in 97 high anxious students.
• Attentional bias training transferred to interpretive bias, but not vice versa.
• This suggests attentional bias has downstream effects on interpretation.

## 1 Introduction

Cognitive models emphasise the critical role that selective processing plays in the onset and maintenance of anxiety (e.g., Beck and Clark, 1997, Eysenck, 1997, Mathews and Mackintosh, 1998, Mogg and Bradley, 1998, Williams et al., 1997). Extensive research generated from these models has shown that anxious individuals disproportionately attend to threat-related stimuli in the environment (attention bias; cf. Bar-Haim, Lamy, Pergamin, Bakermans-Kranenburg, & van IJzendoorn, 2007; Fox et al., 2002, MacLeod et al., 1986) and perceive threat-congruous meanings when processing ambiguous information (interpretation bias; cf. Eysenck et al., 1991, Mathews and MacLeod, 2002). Modifying attentional and interpretive bias using experimental procedures (Cognitive Bias Modification; CBM) has demonstrated the causal role of each of these biases in anxiety (see MacLeod & Mathews, 2012, for a review).

In the CBM paradigm, participants carry out repeated trials in which they are trained to interpret emotional ambiguity in either a negative direction (e.g., Salemink, van den Hout, & Kindt, 2007) or a positive direction (e.g., Beard & Amir, 2008; CBM for interpretation, CBM-I); to attend to threat (e.g., White, Suway, Pine, Bar-Haim, & Fox, 2011), or to attend away from threat stimuli on the computer screen (e.g., Browning et al., 2010, Hakamata et al., 2010; CBM for attention, CBM-A). CBM-I to promote positive interpretation and CBM-A to encourage attention away from threat have been shown to reduce symptoms of anxiety in clinical and high anxious samples (e.g., Amir et al., 2009, Amir and Taylor, 2012, Brosan et al., 2011, Linetzky et al., 2015, Salemink et al., 2009, Schmidt et al., 2009). However, whilst this research is consistent with the theory that the presence of one or more of these biases underpins anxiety, it is not known whether these biases reflect one common neurocognitive mechanism. For example, models such as Mathews and Mackintosh (1998) and Bishop (2007) propose that both attentional and interpretive biases arise from the outcome from competition of bottom-up (a relatively automatic threat evaluation system) and top-down (cognitive control) cognitive processes. As these models predict that both biases arise from the same system, it is possible that modifying the system to alter one bias (e.g., attention), will also impact on the presence of the other bias (e.g., interpretation). Cognitive bias modification paradigms therefore allow us to address such interesting theoretical questions regarding the interacting nature of these two biases, by modifying one bias and assessing impact on the other.

Additionally this can help answer important therapeutic questions, such as whether modifying one bias (e.g., attention bias) is sufficient to alter cognitive processing in other areas (e.g., in interpretation). In order to investigate these ideas, White et al. (2011) trained participants to attend to threat using the dot-probe task and then assessed its impact on a test of interpretive bias. In the training phase, two faces (one angry and one neutral) were presented on the computer screen above and below a central fixation point. After 500 ms they disappeared, were replaced by an arrow pointing either up or down, and participants were required to indicate in which direction the arrow was pointing. In the attend threat training condition the probes consistently replaced the threat-related faces, and in the placebo condition probes replaced threat-related and neutral faces with equal probability. In a subsequent test of interpretation of emotional ambiguity, attend threat training appeared to increase the tendency of participants to make threat-related initial interpretations of emotionally ambiguous sentences.

These results provide initial evidence that attentional and interpretive biases result from a common neurocognitive mechanism, as opposed to being orthogonal. However, the results do not allow us to speculate on the temporal nature of these biases. That is, it might be that biases in attention precede and subsequently influence interpretive bias in a downstream manner (Ouimet, Gawronski, & Dozois, 2009), with the influence of interpretive processing on upstream attentional processing being more difficult. Two studies have tested the impact of modifying interpretive bias on attentional bias (Amir et al., 2010, Mobini et al., 2014). Consistent with a common mechanism hypothesis (in which modifying either bias impacts on a central mechanism related to the other bias) modifying threat-related interpretive bias did have an impact upon threat-related attentional bias.

Although preliminary work appears to be consistent with attentional and interpretive biases sharing a common mechanism, these initial exciting findings clearly need replication. The prior investigation of transfer from attentional retraining to interpretive bias (White et al., 2011) did not assess the impact of therapeutic CBM-A versus a control group, instead retraining attention towards threat. We therefore concentrated on positive (non-threat focussed) CBM (multiple sessions of either CBM-A or CBM-I) in a high anxious sample, enabling us to address the applied question of whether either CBM-A or CBM-I has broader effects on the cognitive biases underpinning anxiety, and as such whether one or the other might be most beneficial to use therapeutically, a question that is currently unanswered. The present study aimed to replicate previous findings that CBM-A modifies interpretive selectivity (White et al., 2011) and CBM-I modifies attentional selectivity (e.g., Amir et al., 2010, Mobini et al., 2014), and develop these findings by testing the comparative transfer effects of neutral CBM on alleviating threat-related attentional and interpretive biases. We sought to further understand the relationship between attentional and interpretive selectivity by training one group of participants to interpret emotional ambiguity in a positive direction (using CBM-I) and another group to focus their attention on non-threat (as opposed to threat-related) stimuli (CBM-A). Two control groups served as a comparison, (placebo training and no training). The placebo training condition alongside a no training control group allowed us to ensure that any effects of training would be unlikely to be due to placebo/demand characteristics. If there were no differences between either the CBM-I/CBM-A groups with the placebo training group, but these three groups all showed improvement relative to our fourth control group (no training), we would be able to determine that the results are likely to be due to a placebo effect or demand characteristics. In line with a common mechanism hypothesis, we predicted that training attention away from threat stimuli would encourage participants to interpret emotional ambiguity in a more positive manner, and that inducing a positive interpretive bias would lead to participants finding it easier to attend away from threat stimuli. In addition to these specific hypotheses, we also predicted that participants in the two training groups (CBM-I and CBM-A) would show reductions in symptoms of anxiety and depression. Finally, we asked participants to keep a diary during the study to assess whether they encountered any major life events throughout the training and to generally assess how they were settling into university life.

## 2 Methods

### 2.1 Participants

Ninety-seven University of East Anglia first year undergraduates (67 females and 30 males, mean age 18.9 years, *SD* = 2.06) were recruited at the start of their first term via emails and a poster campaign. Four eligibility questions emailed to all interested students had determined that participants were native English speakers who felt anxious and/or overwhelmed about starting University, had no difficulties reading or understanding text from a computer screen and had not previously completed a University course. Participants were randomly (with the constraint that group size should be approximately equal) allocated to one of four conditions^1^: attention bias training (*n* = 24; 17 females and 7 males; mean age 19.08 years, *SD* = 2.93), interpretation bias training (*n* = 26; 15 females and 11 males; mean age 18.96 years, *SD* = 2.44), control task training (*n* = 25; 20 females and 5 males; mean age 18.84 years, *SD* = 1.11) and no training (*n* = 22; 15 females and 7 males; mean age 18.68, *SD* = 0.95). Participants in the training conditions were paid £50 for their time and those in the no training control group were paid £24.

### 2.2 Materials

#### 2.2.1 Attention bias test

The attention bias test employed the dot-probe paradigm similar to that of MacLeod and colleagues (MacLeod, Rutherford, Campbell, Ebsworthy, & Holker, 2002), and was administered using E-Prime software (Schneider, Eschman, & Zuccolotto, 2002). The dot-probe assessment consisted of 96 trials (12 word pairs) which were administered pre and post training. In each trial, first a fixation cross was presented in the centre of the screen for 500 ms, followed immediately by a word pair (matched for frequency and length). Each stimulus pair contained one neutral word (e.g., “detailed”) and one threat-related word (e.g., “afraid”). Words were presented onscreen for 500 ms, equidistant above and below the fixation cross 3 cm apart. Next, an arrow probe (“<” or “>” with equal frequency) appeared in the prior location of one of the words until response. Participants were instructed to indicate which direction the arrow was pointing by pressing the left or right arrow key as quickly and as accurately as possible. Arrow probes replaced the different word types (threat/neutral) with equal frequency, and appeared with equal probability above and below the fixation cross. A non-threat, neutral, attentional bias (i.e., an ability to attend away from threat) was indicated by faster reaction times to probes in non-threat word positions (as opposed to probes replacing threat words).

#### 2.2.2 Attention bias modification *CBM-A*

Attention bias training, also administered using E-Prime (Schneider et al., 2002), comprised eight sessions spread over four weeks and was again similar to that used originally by MacLeod et al. (2002) and in line with that used by See, MacLeod, and Bridle (2009). Each session consisted of 384 trials (96 word pairs in total). Training took the same format as in the attention bias test, except that the arrow was always in the position of the neutral word in each pair, with the intention of retraining attention away from the anxiety related material. Participants keyed in the direction of the arrow probe (randomised for each trial), which cleared the screen and initiated the next trial after 1 s. In the first training session the same twelve word pairs were used as in the bias test. In each subsequent training session an additional twelve word pairs were added to the stimulus set, although the number of trials remained the same.

#### 2.2.3 Interpretation bias test

Based on Mathews and Mackintosh (2000), in the interpretation bias test ten emotionally ambiguous vignettes were firstly presented on screen using E-Run software (Schneider et al., 2002). Participants were asked to read and imagine themselves vividly in each situation. Each vignette related to one of two categories, either typical social performance or social interaction situations, for instance: *“The bus ride: You get on a bus and find an empty seat next to one that has a rip in it. At the next stop several people get on and the seat next to you remains vacant.”* Each scenario contained four lines, appearing on screen one at a time when the participant pressed a key indicating they were ready to advance. After each one, participants rated how pleasant they found the scenario on a scale of 1–9 (9 being most pleasant) and how vividly they had imagined themselves in the scenario on a scale of 1–5 (5 being most vivid). Note that we found no significant impact of training on pleasantness ratings, and these are not discussed further. Given the strong relationship between imagery and emotion it is possible that enhanced vividness of imagery could lead to stronger emotional effects (Holmes & Mathews, 2005). We therefore assessed vividness of imagery in order to rule this out. A comprehension question then followed to check participants had read and understood the situation described *(e.g., “Were the people who got on strangers to you?”).* Responses were collected using left and right arrow keys and feedback indicated whether the response had been “Correct!” or “Incorrect”. Feedback was followed immediately by presentation of a new titled scenario. The order in which scenarios were presented was conserved across all participants.

After the ten vignettes had been presented, interpretation bias was measured using the recognition test. In this test, the title of the first scenario was presented along with four unambiguous statements related to each scenario (presented one at a time). Participants rated how similar each statement was in meaning to the scenario with that title, on a scale of 1–4 (4 being very similar in meaning). Two of the four statements were foils that were not possible interpretations and two were target items that were possible interpretations of the scenario. For each scenario, one target and one foil statement described a positive outcome (e.g., *“The seat next to you remains empty because it is damaged” and “The person in the seat next to you talks to you in a friendly way” respectively)* and one target and one foil statement described a negative outcome *(*e.g., *“The seat next to you is empty because no one wants to sit with you”* and *“The person in the seat next you makes a rip in the fabric” respectively).* In this task, higher similarity ratings for negative targets as compared to positive targets reflects a more negative interpretive bias. The foil scenarios allow an understanding of whether the intervention has led to a change in interpretation per se, or a more general response bias to respond differently to positive/negative material. Order of presentation of the four recognition statements was randomised. There were two versions of the interpretation bias test which were identical in structure and theme but contained different scenarios. Each participant completed one version at baseline and the other version at post-training (order was counterbalanced across participants).

#### 2.2.4 Interpretation bias modification *CBM-I*

The interpretation bias training was based on the method used originally by Mathews and Mackintosh (2000). Each of the eight CBM-I sessions, also administered using E-Prime (Schneider et al., 2002), comprised 21 scenarios (with new scenarios in each session) which participants read on screen. They related to concerns that new students might have, including homesickness, financial, academic and social concerns. Each scenario had the final word missing and was ambiguous up to this point *(*e.g., *“A new course tutor is appointed for your history class and you hear that they are very disciplined and hard working. When you meet them for the first time to discuss your interests, you think that your tutor found your work ----”)*. The final word always resolved the ambiguity in a positive way, and was presented in an incomplete form on the screen after the participant had read the preceding scenario *(*e.g., *th-r---h “thorough”).* The participants’ task was to use the preceding scenario to create an image and use this to identify the incomplete word, and type in the first missing letter. A comprehension question was then shown that related to an interpretation of the scenario *(*e.g., *“Did your new tutor have a bad opinion of your work?”)*. Participants used the arrow keys to answer “Yes” or “No” to this question, followed by feedback (a *“Correct!”* or *“Incorrect”* message). There was no overlap in the materials used for interpretive training and interpretive bias assessment.

#### 2.2.5 CBM-placebo training

These were exactly as for the attentional CBM-A training group, except that household words were used instead of anxiety-related words. Each household word was in a pair with a matched non-household neutral word, and the arrow probe was distributed with equal probability between word types.

#### 2.2.6 Anxiety and depression questionnaires

Participants completed self-report measures of anxiety and depression at baseline, post-training and two-week follow-up. Social anxiety and trait anxiety were assessed using the Fear of Negative Evaluation scale (FNE; Watson & Friend, 1969) and trait scale of the Spielberger State-Trait Anxiety Inventory (STAI-T; Spielberger, Gorsuch, Lushene, Vagg, & Jacobs, 1983), respectively. Depression was measured using the Beck Depression Inventory 2nd Edition (BDI-II; Beck, Steer, & Brown, 1996).

#### 2.2.7 Weekly diary and feedback questionnaires

A weekly diary questionnaire was developed to check how students were coping with and adjusting to University life. The questions related to social life, family contact, health, academic attendance and performance, homework deadlines, meetings with advisers and paid employment and consisted of a mixture of open ended questions and ratings. Two feedback questionnaires, also developed for the present research, probed how participants had found their experience of the study. These were included for practical reasons to gain feedback on participants’ experience during the course of the study, and no formal analysis of data from these is presented.

### 2.3 Procedure

#### 2.3.1 First test session (baseline)

Ethical approval for the study was obtained from the University of East Anglia School of Social Work and Psychology Research Ethics Committee. Participants were informed on an information sheet that we were interested in finding ways in which we could help make the transition to university easier and were not given any further indication regarding how the tasks might be working to help them. After written consent had been given, eligible participants completed the first session of the study at which baseline measures of attentional bias, interpretive bias, fear of negative evaluations, trait anxiety and depression were taken. Following this, participants in the no training group were thanked and asked to return to the psychology laboratory in four weeks. All other participants performed a practice version of the training task specific to their group in preparation for the training sessions that would form the next part of the study.

#### 2.3.2 Training sessions: CBM-A, CBM-I and CBM-placebo training

Participants completed eight training sessions specific to their condition in the laboratory. They were instructed to complete two half-hour sessions per week for four weeks, with the sessions always being on separate days, although in practice this schedule was not always possible (see *Participants* section of Results). Two participants completed training sessions back to back (i.e., on the same day). Three participants missed one training session (one from CBM-I, one from CBM-A and one from CBM-placebo). One further participant from the CBM-placebo condition missed two training sessions. During the four week training period all participants (including the no training control group) were sent the weekly diary questionnaire once a week, which they subsequently filled in and returned via email.

#### 2.3.3 Second and third test session (post-training and follow-up)

The second test session (post-training) was scheduled to take place two to seven days after completion of the eighth training session, and four weeks after the first test session for the no training control group. It included measures of attentional bias, interpretive bias, fear of negative evaluations, trait anxiety and depression. The third (follow-up) session of the study took place two weeks after the post-training session and included only an assessment of fear of negative evaluation, trait anxiety and depression, and debriefing after the feedback questionnaires had been completed.

## 3 Results

### 3.1 Participants

Three participants were excluded from the analysis. Two participants (one CBM-A; one control training) experienced significant life events and had excessive gaps between sessions (as reported in their weekly diary questionnaires), and one (CBM-A) exhibited high levels of inaccuracy in attention bias training sessions (>3 *SD* from group mean). The descriptive statistics for age, gender and baseline trait anxiety, depression and fear of negative evaluation are presented in Table 1. All groups were comparable at baseline on all measures of anxiety, depression, attention bias and interpretation bias (all *Fs* < 1.5, ns).

Overall, the sample (*N* = 94) had a mean trait anxiety score of 48.40, which is approximately one standard deviation above the STAI-T reported norms and equivalent to around the 78th percentile for University students (Spielberger et al., 1983). They had a mean BDI-II score of 14.98, indicative of mild depression levels (Beck et al., 1996), and a mean FNE score of 20.17 corresponding with scorers who are sometimes fearful in social-evaluative situations (Watson & Friend, 1969).

### 3.2 Impact of CBM on attentional bias

In keeping with the approach adopted by MacLeod et al. (2002), median RTs to the probe following each word type (threat-related and neutral) were calculated for every participant and RTs were excluded if they were less than 200 ms and greater than 2000 ms. Using the mean plus or minus three times the standard deviation approach, one participant was found to have outlying attention bias scores (RT to threat-related words minus RT to neutral words in milliseconds) reflecting a very negative bias at baseline and a very positive bias in the post-training session; these values were replaced with the next extreme plus one (Tabachnick & Fidell, 2001). All four groups averaged 94% accuracy (number of times the correct probe direction was keyed) on the attention bias test in both the baseline and post-training sessions; two participants who had low accuracy within a single session (control task group, 82% accuracy, baseline; no training group, 80%, post-training) were retained in the analysis. To assess change in bias over the training period, an attentional bias index was calculated by subtracting the median reaction time (ms) to neutral words from the median reaction time (ms) to threat-related words. Therefore, a negative score reflected participants showing a more threat-related attentional bias.

**Fig. 1:**
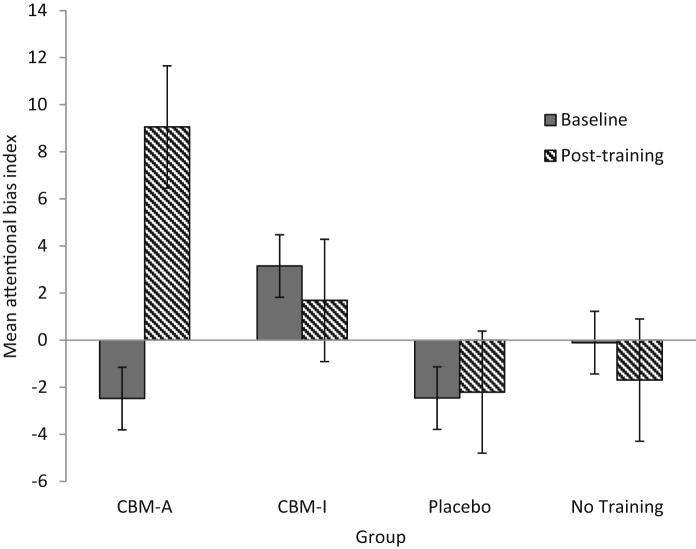
Mean attentional bias index at baseline and post-training (a more positive score indicates a more positive attentional bias). Error bar represent ± 1 standard error.

A one-way ANOVA confirmed there had been no significant differences between groups in baseline attention bias index, *F* (3, 90) = 1.01, *p* = 0.39; see Fig. 1. A mixed model ANOVA with time (baseline, post-training) as the within participants factor and group (CBM-A, CBM-I, CBM-placebo or no training) as the between subjects factor was conducted on the attentional bias index data. No main effect of time was found, *F* (1, 90) = 1.08, *p* = 0.30, η_p_^2^ = 0.01. There was, however, a trend-level time by group interaction, *F* (3, 90) = 2.16, *p* = 0.099, η_p_^2^ = 0.067. Because of our specific predictions that both attention bias training and interpretation bias training would influence attention bias, but that the control groups would not, we conducted paired sampled t-tests within each group to examine changes in attention bias scores from baseline to post-training. The only significant finding was of CBM-A, *t* (21) = −2.08, *p* = 0.025, (one-tailed), *r* = 0.42, indicating that as predicted the attentional training resulted in a significant reduction in threat-related attention bias from pre-training to post-training. Contrary to prediction, the CBM-I group did not generate a significant reduction in attention bias, *t* (25) = 0.44, *p* = 0.34 (one-tailed), and any alterations in bias in both control groups were similarly non-significant, *t*s < 1 (see Fig. 1)^2^.

### 3.3 Impact of CBM on interpretation bias

One participant who due to computer error did not complete the second interpretation bias test could not be included in the analysis. Change in interpretation bias over the four weeks was assessed using a mixed model ANOVA with time (baseline, post-training), scenario category (social interaction, performance), sentence type (foil, target) and sentence valence (positive, negative) as the within subjects variables and training group as the between subjects factor. There was a main effect of time, *F* (1, 89) = 4.06, *p* = 0.047, η_p_^2^ = 0.04, indicating there had been an overall increase in positive interpretation bias, and of category, *F* (1, 89) = 73.39, *p* < 0.001, η_p_^2^ = 0.45, with performance sentences being endorsed more than social interaction sentences. There was also a main effect of type, *F* (1, 89) = 7.72, *p* < 0.001, η_p_^2^ = 0.90, suggesting greater endorsement of possible interpretations of the scenarios than of foils, and valence, *F* (1, 89) = 48.39, *p* < 0.001, η_p_^2^ = 0.35, with positive sentences being selected more than negative ones. Crucially, however, these effects were qualified by a significant time by valence by group interaction, *F* (3, 89) = 3.55, *p* = 0.018, η_p_^2^ = 0.11, and a significant time by type by valence by group interaction, *F* (3, 89) = 3.21, *p* = 0.027, η_p_^2^ = 0.10.

To examine the four-way interaction of time by type by valence by group more closely, separate analyses were conducted on target sentences (i.e., possible interpretations of the scenarios) and foil sentences. On targets, there was a significant time by valence by group interaction, *F* (3, 89) = 3.88, *p* = 0.012, η_p_^2^ = 0.12, whereas on foils, the time by valence by group interaction was non-significant, *F* (3, 89) = 1.57, *p* = 0.20.

**Fig. 2:**
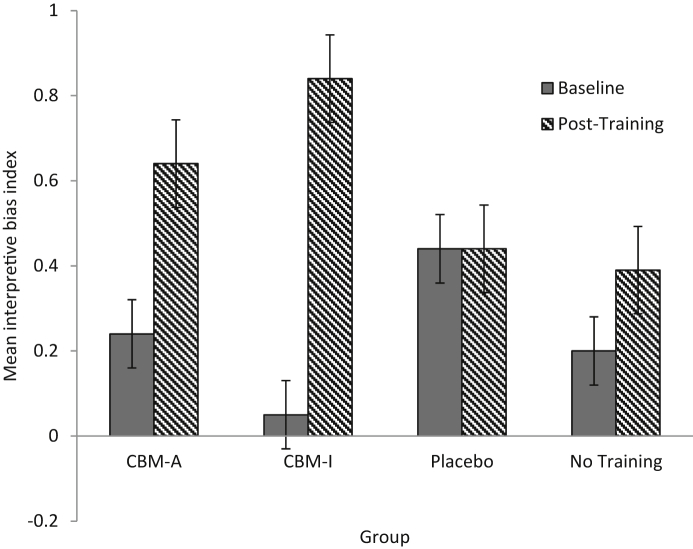
Mean interpretive bias index at baseline and post-training (a more positive score indicates a more positive interpretive bias). Error bars represent ± 1 standard error.

To further explore the time by valence by group interaction on target sentences, an interpretation bias index was calculated by subtracting the mean recognition ratings for negative target sentences from the mean recognition ratings for positive target sentences. A mixed model ANOVA revealed a significant main effect of time, *F* (1, 89) = 14.94, p < 0.001 η_p_^2^ = 0.14, qualified by a significant group by time interaction, *F* (3, 89) = 3.89, *p* = 0.012, η_p_^2^ = 0.12. Paired sample t-tests conducted on the interpretation bias test data revealed that both the attention training group, *t* (21) = −2.04, *p* = 0.027 (one-tailed), *r* = - 0.41, and the interpretation training group, *t* (25) = −5.20, *p* < 0.001 (one-tailed), *r* = 0.72, displayed a significantly more positive interpretation bias at post-training than baseline. The change in interpretation bias did not reach significance in the control training group, *t* (22) = 0.024, *p* = 0.49 (one-tailed), or no training group, *t* (21) = −0.91, *p* = 0.19 (one-tailed; see Fig. 2).

A separate mixed model ANOVA was conducted on the ratings of how vividly each scenario was imagined, with time (baseline, post-training) and category (social interaction, performance) entered as the within subjects factors and group entered as the between subjects factor. This revealed a main effect of category, *F* (1, 89) = 29.34, *p* < 0.001, η_p_^2^ = 0.25, and a time by category by group interaction, *F* (3, 89) = 2.79, *p* = 0.045, η_p_^2^ = 0.086. Follow-up paired sample t-tests suggested that the only significant change was in the control training group who had imagined performance situations significantly more vividly at post-training (*M* = 3.30, *SD* = 0.75) than baseline (*M* = 2.91, *SD* = 0.72), *t (*22) = −3.27, *p* = 0.004, *r* = 0.57.

### 3.4 Further analysis of asymmetrical transfer effects

To further explore the apparent asymmetrical transfer effect (where CBM-A generalises to interpretive bias, but CBM-I does not generalise to attentional bias) positive bias increase scores were computed to directly compare improvement in interpretive bias with improvement in attention bias for the CBM-A and CBM-I groups only. These increase scores were then converted to z-scores so change in each bias could be directly compared. A mixed model ANOVA was run with bias (attention or interpretation) as the within groups factor and group (CBM-A, CBM-I) as the between groups factor. Results indicated a significant interaction between bias and group, *F* (1, 46) = 6.61, *p* = 0.013, η_p_^2^ = 0.13. Planned comparisons suggested that for attention training there was no differential impact on attention versus interpretive bias, *t* (21) = 1.17, *p* = 0.26. For interpretive training the effect on interpretive bias was clearly stronger than the effect on attentional bias, *t* (25) = - 2.72, *p* = 0.012, *r* = 0.48.

### 3.5 Impact of CBM on self-report measures of anxiety and depression

One participant in the no training group whose scores on the BDI-II were above three standard deviations from the mean at baseline and post-training were replaced with the next extreme plus one (Tabachnick & Fidell, 2001). Separate mixed model ANOVAs (one for each outcome measure) were performed on the data, each with time (baseline, post-training, follow-up) as the within subjects variables and group (CBM-A, CBM-I, placebo training or no training) as the between subjects variable. Results revealed a main effect of time (*p* < 0.001) for all three measures, indicating all participants felt better as the study progressed, but significant time by group interactions were not found for trait anxiety, *F* (4.73, 141.7) = 1.26, *p* = 0.29, or fear of negative evaluation, *F* < 1, see Table 2. For depression, results indicated a near significant interaction, *F* (4.84, 145.2) = 2.13, *p* = 0.068, η_p_^2^ = 0.066. Follow-up LSD tests however indicated that the differences in depression reduction between groups had been non-significant, *ps* > 0.1, see Table 2.

## 4 Discussion

The results demonstrate that both methods of cognitive bias modification (attentional and interpretive) can successfully modify cognitive responding within the trained domain. Interpretation bias test findings clearly demonstrated that positive interpretive training enhanced positive interpretation bias, and, although the attention bias test time by group interaction was at trend-level significance, further analyses indicated that attention bias training to avoid threat had modified attentional bias in the expected direction. Crucially, in addition the present study suggests that training attention away from threat (CBM-A) transfers across a cognitive domain, influencing subsequent interpretation of emotional ambiguity, with a substantial increase in positive interpretive bias evident in the attentional training group. Previous research (White et al., 2011) has only demonstrated this transfer effect with attentional training to attend to threat; the present results suggest that in high anxious individuals, attentional training to avoid threat (and attend to non-threat stimuli) could impact on both their attentional and interpretive bias. However, contrary to previous research (Amir et al., 2010, Mobini et al., 2014), the demonstrated transfer effects were not reciprocal, and interpretive training (CBM-I) did not aid attentional avoidance of threat stimuli over neutral stimuli.

The findings that modifying attentional bias has an impact on interpretive bias are in line with current models which propose biased attentional and interpretive processes are not orthogonal and instead result from a single common mechanism (e.g., Bishop, 2007, Mathews and Mackintosh, 1998). However, these models might also predict that modifying interpretive bias should impact upon attentional bias (given the shared mechanism underlying them). Although there is some evidence suggesting this to be the case (Amir et al., 2010, Mobini et al., 2014), we were not able to replicate this finding in the present study. This could be due to methodological differences between the studies. For example, whereas in the present study the post-training assessment of attentional bias took place two to seven days after the final CBM session, in both of the prior studies effects on attentional bias were apparent only immediately following a single session of CBM-I (Amir et al., 2010, Mobini et al., 2014). One of the studies also measured attentional bias at one-week post CBM-I, when effects were no longer evident (Mobini et al., 2014). Nonetheless, the discrepancy in findings suggests further work is clearly needed. For example, it is possible that attentional training might at least have broader or more easily generalisable cognitive effects as compared to interpretive training, perhaps due to additional downstream impacts upon interpretive bias.

Although in the present experiment CBM successfully modified attentional and interpretive processing of threat-related information, it did not impact on either trait anxiety, depression or fear of negative evaluation over and above our control conditions (all groups showed significant decreases on self-report measures of anxiety, fear of negative evaluations and depression). This appears to be in contrast to previous studies that have shown successful use of CBM to reduce high anxiety in non-clinical populations (e.g., Beard & Amir, 2008; Murphy et al., 2007; Salemink et al., 2009). There are three possible explanations for this unexpected finding. First, it is possible that the current intervention did not provide enough training to be effective (for example, there were only 21 training trials in each session for the interpretive training), and that by increasing the “dosage” the effects of CBM on self-report anxiety and depression might become apparent. Second, the absence of any effects on anxiety and depression could have been due to the fact that our participants did not start with clearly negative cognitive biases. For example, across the groups the mean scores suggest a relatively more positive interpretive bias at baseline. This could be problematic in terms of the success of CBM given that research suggests that having a more threat-related bias at baseline is linked to greater symptom reduction (Amir, Taylor, & Donohue, 2011). Third, in the present study students did not complete the training program prior to the real-world stressor, instead beginning training after they had arrived at University. This is a crucial difference between the current study and previous research which has suggested, contrary to the present findings, that CBM for attention can attenuate anxiety associated with starting university as compared to a no training control group (See et al., 2009). In their study, See et al. (2009) asked participants to take part in the training program prior to starting at university (which was overseas), then anxiety was assessed during the subsequent stressor (on arrival in the country and 48 h after the final training session). In the current experiment students were asked to take part in the training program during the stressor, training began once term had started and the post-training measurement of anxiety, fear and depression occurred four weeks later. Whereas See et al. (2009) found that anxiety had increased over training in both their groups (CBM-A and control), this increase was attenuated in the CBM-A group. In the current experiment all groups showed relatively large decreases in anxiety, fear and depression over time (which might have occurred naturally, or perhaps due to some therapeutic effect of carrying out the weekly diary task in the present study). Hence, in comparison with the previous finding, it seems that commencing training after all first year undergraduate students had successfully moved onto campus and started their University career failed to capture anxiety reactivity to the stressful event itself.

Although an effect on anxiety reactivity was not demonstrated, the finding that training attention away from threat reduces threat-related interpretation bias might have important therapeutic implications. Past research has indicated that severe threat-related attention bias may predict poor treatment response to Cognitive Behavioural Therapy (CBT), commonly used in the treatment of anxiety (Legerstee et al., 2009, Rachman, 2015). The results of the present study and previous research (White et al., 2011) suggest that it would be beneficial for negative attention bias to be targeted with modification procedures prior to commencing CBT. The broad cognitive effects of attention bias modification indicate that this might facilitate downstream cognitive restructuring, potentially enhancing CBT efficacy. Hence attention bias modification could be used as an at home waiting list treatment (Brosan et al., 2011), or alternatively as an adjunct therapy (Amir and Taylor, 2012, Linetzky et al., 2015, Shechner et al., 2014). The present experiment tentatively suggests that attentional training alone might be more beneficial in a therapeutic sense than interpretive training alone (due to the transfer across cognitive biases). However, given the discrepant findings in the literature (the contrast between the present study and, Amir et al., 2010, Mobini et al., 2014) it is too early to conclude this, and further research is clearly warranted.

Current findings should be interpreted in view of several limitations not previously mentioned. First, the factorial design entailed that, whilst 97 participants completed the study, only one quarter of this sample was allocated to each group. Reverse transfer effects (from CBM-I to attention) may have been discernible had group sizes been larger. Second, the nature of any CBM-A effects on attentional bias is subject to the reliability of the visual-probe task, criticised by some commentators (e.g., Staugaard, 2009). Whilst it would certainly be beneficial for future research to examine how bias modification and assessment can be optimised (e.g., Clarke, Notebaert, & MacLeod, 2014; MacLeod & Clarke, 2015), the present effect of CBM-A (although a trend, statistically reliable when baseline bias was held constant) on attentional bias combined with its far transfer effects to interpretive bias suggest that it was sufficiently robust to impact on both processing modalities. Nevertheless, replication of findings is needed before firm conclusions can be drawn. The post hoc finding that the impact of CBM on alleviating threat bias was affected by pre-existing processing selectivity suggests it might be important in future work to consider more closely the bias profile of participants at baseline, and how this relates to subsequent change in threat bias and symptom outcomes (cf. e.g., Fox et al., 2015; MacLeod & Clarke, 2015; Maoz et al., 2013; Mogoaşe et al., 2014). Third, it would have been preferable for the attentional bias test to have included words that were not used in the retraining procedure. Use of the same stimuli across tasks leaves open the possibility that the observed impact of CBM-A on attention reflected a learned attentional response to the specific training words, rather than modified attention to the target emotional class of information. However, the fact that CBM-A transferred to a measure of interpretive bias suggests that it induced more than a simple trained response to particular words employed within the program. There is also good evidence from other studies using different stimuli that CBM-A modifies threat-related attentional selectivity (e.g., Hakamata et al., 2010). Fourth, the control task for CBM-A and CBM-I would ideally have utilised sham training (i.e., applied the same stimuli as in each of the active programs, without the training contingency) to mitigate the possibility that training effects were due to differential exposure to emotional information and not the intended attentional/interpretive mechanisms. This would have led to three control groups, however, which was not possible given our pool of potential participants. The present CBM-placebo program was intended to control for taking part in a computer task in the laboratory on multiple occasions. Fifth, although selected as a widely used measure of interpretive bias that has established sensitivity in detecting threat bias change (e.g., Mobini, Reynolds, & Mackintosh, 2013), the recognition task may be criticised for not being process-pure as the similarity ratings (from which the bias index is derived) are based on participants’ recollections of the earlier presented scenarios. This leaves unclear whether attentional retraining affects early, spontaneous interpretive selectivity or later more elaborative processes involved in the retrieval and assimilation of content (cf. Rusu & Pincus, 2012). Future research could address this question using different measures of interpretive bias.

In conclusion, we found that when participants completed a four week programme of CBM-A, threat-related attention bias was alleviated and interpretation bias became more positive, in a group of participants who had self-reported anxiety about starting University. This result suggests that CBM-A might have broad cognitive effects if used in a therapeutic setting. In contrast, we failed to replicate previous research which has suggested the reverse is also true (Amir et al., 2010, Mobini et al., 2014). In the present study, participants completing a four week course of CBM-I did not exhibit similar transfer of effects to the untrained attentional domain.

## Figures and Tables

**Table 1:** Mean age, trait anxiety (STAI-T), depression (BDI-II) and fear of negative evaluations (FNE) with standard deviations and gender ratio.

	CBM-A (*n* = 22)		CBM-I (*n* = 26)		CBM-placebo (*n* = 24)		No training (*n* = 22)		F test
	M	SD	M	SD	M	SD	M	SD	
Age	19.14	3.06	18.96	2.34	18.83	1.13	18.68	0.95	0.19
Female:Male	15:7		15:11		19:5		15:7		
STAI-T	48.18	9.08	47.12	8.58	50.25	9.19	48.14	10.38	0.49
BDI-II	14.64	6.92	13.65	8.40	17.54	7.32	13.73	8.15	1.33
FNE	21.57	6.42	18.08	6.95	20.38	6.91	21.09	7.93	1.17
Attention bias	−3.86	15.19	3.15	12.00	−2.46	14.78	−0.11	14.16	1.17
Interpretation bias	0.24	0.55	0.05	0.64	0.44	0.82	0.20	0.59	1.46

Note: all F values were non-significant (p > 0.1).

**Table 2:** Mean self-reported trait anxiety (STAI-T), fear of negative evaluations (FNE), and depression scores (BDI-II) at baseline, post-training and follow-up.

	Baseline (pre-training)			Post-training			Follow-up		
	STAI-T	FNE	BDI-II	STAI-T	FNE	BDI-II	STAI-T	FNE	BDI-II
CBM-A	48.18 (9.08)	21.57 (6.42)	14.64 (6.92)	45.68 (7.71)	20.27 (6.78)	12.95 (5.69)	45.14 (8.26)	18.45 (8.11)	11.59 (5.53)
CBM-I	47.12 (8.58)	18.08 6.95)	13.65 (8.40)	42.46 (7.07)	16.08 (9.13)	10.81 (6.66)	41.42 (9.64)	14.77 (9.18)	8.04 (5.43)
CBM-placebo	50.25 (9.19)	20.38 (6.91)	17.54 (7.32)	44.29 (9.55)	17.42 (8.04)	11.50 (5.32)	42.42 (8.87)	16.63 (8.04)	11.50 (6.19)
No training	48.14 (10.38)	21.09 (7.93)	13.73 (8.15)	43.68 (11.88)	18.14 (8.29)	11.64 (9.31)	41.82 (12.08)	17.59 (8.42)	9.73 (9.43)

*Note:* standard deviations in parentheses.

## Footnotes

1 It should be noted that participants for this study were recruited at the beginning of two academic years (2008 and 2010). At the start of the first academic year of testing, participants were randomly allocated to CBM-A (*n* = 12), CBM-I (*n* = 13) and the placebo control condition (*n* = 15). At the start of the second academic year of testing participants were randomly allocated to CBM-A (*n* = 12), CBM-I (*n* = 13), placebo control condition (*n* = 10) and no training control (*n* = 22) with the restriction that group sizes would be approximately equal.

2 The failure to find the predicted significant difference between the CBM-A group and the control groups for attention bias raises the possibility that the intended attentional bias modification was not achieved. There is growing evidence that CBM may be particularly efficacious for participants who have a pre-existing cognitive bias to threat, but be less beneficial for individuals with no pre-existing bias (e.g., Fox et al., 2015, Maoz et al., 2013, Mogoase et al., 2014). This could result in a reduction in statistical power (hence the trend-level finding for attention) if the intervention affected participants differently based on their pre-existing bias scores. Since levels of attentional and interpretive bias did not differ significantly between groups (Table 1), we decided to rerun analyses with baseline attentional/interpretive bias included as a covariate. For attention, there was a significant effect of baseline attention bias, *F* (1, 89) = 129.21, *p* < 0.001, η_p_^2^ = 0.59, and a significant effect of group on change in attention bias, *F* (3, 89) = 3.46, *p* = 0.02, η_p_^2^ = 0.11. For interpretation, there was a significant effect of baseline interpretive bias, *F* (1, 89) = 44.51, *p* < 0.001, η_p_^2^ = 0.33, and a significant effect of group on change in interpretive bias, *F* (3, 89) = 2.87, *p* = 0.041, η_p_^2^ = 0.09. Overall, these findings suggest that CBM-A reliably modified attention bias when the effect of baseline bias was removed from the analysis. CBM-I was similarly successful in modifying interpretation bias.
